# Comparison of two storage methods for flexible, thermolabile endoscopes: is there a difference in microbiological contamination?

**DOI:** 10.1186/2047-2994-4-S1-P55

**Published:** 2015-06-16

**Authors:** D Scalia-Perreard, C Landelle, A Gayet-Ageron, AP Dos Santos, C Mounir, D Pittet

**Affiliations:** 1 Service Prévention et Contrôle de l'Infection, Hôpitaux Universitaires de Genève, Switzerland; 2 Service d'Epidémiologie Clinique, Hôpitaux Universitaires de Genève, Switzerland; 3 Plateau Technique d'Endoscopie, Hôpitaux Universitaires de Genève, Switzerland; 4 Soins Intensifs adultes, Hôpitaux Universitaires de Genève, Switzerland

## Introduction

Currently no recommendations exist to help choose between the drying and storage cabinets for heat-sensitive endoscopes (SCHE) and standard non-ventilated cupboards. We hypothesize that use of the SCHE helps reduce microbiological contamination compared to the standard non-ventilated cupboards.

## Objectives

To compare the level of microbiological contamination of endoscopes stored using the two different methods.

## Methods

Comparative observational study conducted within the HUG (Hôpitaux Universitaires de Genève) between February and July 2014 using flexible bronchoscopes utilized in adult intensive care, stocked in SCHE, and flexible bronchoscopes utilized in the endoscopy equipment tray, stored in standard, non-ventilated cupboards. Samples were taken on day 0 (removal from the disinfecting washer), Day 3 (primary outcome), and Day 7. The samples were collected from endoscope extremities and analyzed after filtration using Millipore^®^ membranes 0.45 µm deposited on blood agar and incubated at 37°C. Microorganisms were identified and numbered and results expressed in the form of colony forming units (CFU) per endoscope.

## Results

In total, 60 samples were collected from 10 bronchoscopes at Day 0, Day 3, and Day 7. No germ was identified among samples taken at Day 0 or Day 3. Among Day 7 samples no germ was identified on endoscopes stored in the standard non-ventilated cupboards, 1 CFU of *Staphylococcus warneri* was identified on a bronchoscope stored in a SCHE (contamination certain).

## Conclusion

We found no microbiological difference between the bronchoscopes stored in ventilated and unventilated cupboards. Only one contaminant was detected at Day 7 on a bronchoscope stored in the SCHE. In spite of the small number of samples, current storage practices utilized at HUG are not brought into question – as long as the retreatment process is adequately followed during the early steps of manual disinfection followed by disinfection in the disinfecting washer.

## Disclosure of interest

None declared.

